# Monitoring the Response of *Plasmodium vivax* to Chloroquine and Uncomplicated *P. falciparum* to Artesunate-fansidar Antimalarials in Southeastern Iran

**Published:** 2018

**Authors:** Hamid AZARIAN MOGHADAM, Mehdi NATEGHPOUR, Ahmad RAEISI, Afsane MOTEVALLI HAGHI, Gholamhosein EDRISSIAN, Leila FARIVAR

**Affiliations:** 1. Dept. of Medical Parasitology & Mycology, School of Public Health, Tehran University of Medical Sciences, Tehran, Iran; 2. Center for Research of Endemic Parasites of Iran, Tehran University of Medical Sciences, Tehran, Iran; 3. Center for Disease Control and Management, Ministry of Health & Medical Education, Tehran, Iran

**Keywords:** Monitoring, *Plasmodium falciparum*, *Plasmodium vivax*, MPCT, Iran

## Abstract

**Background:**

For many years, malaria was a major life-threatening parasitic infection in Iran. Although malaria elimination program is implementing in the country, still some cases annually are reported from malaria-endemic areas.

**Methods:**

This study was conducted in five malaria endemic districts of Sistan and Baluchistan Province, southeastern Iran, neighboring Afghanistan and Pakistan countries. Overall, 170 and 38 *vivax* malaria and *falciparum* malaria infected patients were enrolled in the study from 2013–2014. All the cases were selected according to criteria of the WHO guideline for in vivo drug sensitivity tests in malaria parasites. Evaluation of drug sensitivity test was conducted with some modifications.

**Results:**

The patients with vivax malaria responded to the regimen of chloroquine in 37.4(±15.9), 40(±13.8) and 42(±17.7) h for Pakistani, Iranian and Afghani nationalities respectively based on MPCT evaluation. The results showed a considerable difference between them and Iranian subjects. MPCT for the patients with falciparum malaria was calculated as 28(±18.05), 26(±12.03) and 36(±16.9) h for Iranian, Pakistani and Afghani nationalities respectively. There was a marginally significant difference between Afghani and other nationalities and between males and females.

**Conclusion:**

Treatment of all the patients resulted in ACPR and MPCT of *P. vivax* showed that the parasite became more sensitive to chloroquine than previous years in studied areas.

## Introduction

**Fig. 1:**
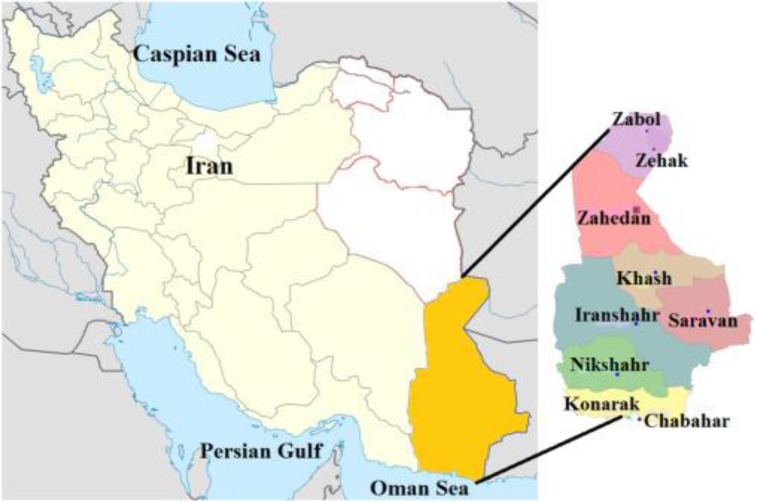
The map of studied areas, Iran

For many years, malaria was a major life-threatening parasitic disease in Iran. Although malaria elimination program is implementing in the country, still some cases annually are reported from malaria-endemic areas. According to the report of Center for Communicable Disease Management (CCDM) in Iran, 1257 cases were recorded in 2014 including 1116 (88.8%), 117 (9.3%) and 23 (1.9%) *P. vivax*, *P. falciparum* and mixed cases respectively (1), that most of them came out from Sistan and Baluchistan and Hormozgan Provinces located in southeastern Iran (Fig. 1).

Moreover, 643 cases were Iranian and the rest non-Iranian. Twenty percent of the cases were recorded as indigenous cases (1). However, some obstacles such as extending drug resistance in human plasmodia, asymptomatic malaria, and imported cases can decelerate implementation of malaria elimination program. Drug resistant phenomenon in *P. falciparum* and more recently in some strains of *P. vivax* (2) against currently used antimalarial drugs can seriously threaten implementation of the program. Indeed, precise and regular monitoring of efficacy of antimalarials against Plasmodia parasites can strengthen the malaria policymakers to overcome such problem.

At present, chloroquine (CQ), a 4-aminoquinoline antimalarial, is the drug of choice for the treatment of *P. vivax* in the most of malarious areas in the world (3,4), but some reports about CQ-resistance in *P. vivax* (2) can make unclear the future of vivax malaria treatment with the drug. On the other hand, although uncomplicated *P. falciparum* is still sensitive to combination of artesunate-fansidar in the most malarious countries in the world, regular in vivo monitoring efficacy of the combination against *P. falciparum* can forecast future situation of the treatment. Results of the preliminary assessments of drug susceptibility in *P. falciparum* and *P. vivax* in Iran were released in 1985 and 1999, respectively (5, 6). Such study was followed by more investigators on occasion in the country (7–11).

This study was proposed to evaluate the susceptibility of *P. vivax* and *P. falciparum* to chloroquine and artesunate-fansidar, currently used antimalarial drugs, after a few years of the last in vivo evaluating antimalarial drugs in Iran.

## Materials and Methods

This study was conducted in Konarak, Chabahar, Sarbaz, Rask, Souran, Mehrastan and Saravan districts of Sistan and Baluchistan Province, Iran. Although location of the province is in southeastern Iran neighboring Afghanistan and Pakistan countries with 300 and 978 km joint borderline respectively, the studied districts are near the borderline of Pakistan. The weather in Chabahar and Konarak is warm and humid with average temperature of 30.2 °C and in the rest, districts warm and low humid with average temperature of 31 °C. *P. vivax* and *P. falciparum* were prevalent species in the areas with dominance of *P. vivax* (1). Recruitment period of the study was covered four transmission seasons (Mar 2013–Feb 2015).

**Patients:** A total of 170 *vivax* malaria infected patients according to the input criteria for sample size estimation [ 95% confidence level, SD of 20 and Precision(d) of 3 h for mean of parasite clearance time (MPCT) of *P. vivax*] were registered for the study, but 33 (19.4%) of them did not complete the study. All 38-falciparum malaria infected patient those referred to health centers of the districts were considered for the study. All patients aged more than 6 months and had the following criteria of WHO guideline: malaria positive *P. vivax* or *P. falciparum* with parasitaemia of 250–100000 parasites/μl blood, axillary temperature equal or more than 37.5 °C or a history of fever during the last 24 h, ability to come for the stipulated follow-up visit. Patients with following criteria were excluded from the study; severe malnutrition, pregnancy, febrile disease other than malaria or other known underlying chronic or severe disease, severe or complicated malaria cases, children aged under 6 months, mixed or mono infections with another *Plasmodium* species detected by microscopy, regular medication which may interfere with antimalarial pharmacokinetics and history of hypersensitivity reactions or contraindications to any of the medicine(s) being tested or used as alternative treatment(s) (9,11, 12). Most of the vivax and falciparum malaria-infected patients were male with 110 and 32 cases respectively. Out of 137 *P. vivax*-infected patients, 91, 43 and 3 cases were Iranian, Pakistani and Afghani. Similarly, out of 38 *P. falciparum*-infected patients 17, 19 and 2 cases were alike the above-mentioned nationalities. Biodata and results of microscopy examination on day 0 were recorded in an appropriate form.

At the enrollment time, a consent form was signed by each of the patients or their guardians. Implementation of the study was approved by TUMS Research Committee under the code number 92-02-160-23635.

**Study techniques:** For evaluation of drug sensitivity in *P. vivax* and *P. falciparum,* two in vivo tests were employed according to the WHO guideline with some modifications (12) as follows:

***P. vivax:*** The included patients were treated with 25mg/kg of chloroquine (CQ) [Parsdaruo co., Iran] divided into three doses over 3 d (10mg/kg on days 0 and 1, and 5mg/kg in day 2). Moreover, to achieve a radical cure amount to 0.75 mg/kg primaquine was administered weekly for 8 wk starting from day 2. Prior to treating the patients, a Giemsa stained thick and thin blood smear was prepared from each of the patients. The asexual parasites were counted against at least 200 WBCs on days 0, 1, 2, 3, 4, 7, 14, 21& 28 in the thick blood smears and then converted to the number of parasites per microliter of blood. Results of the counting, to understand drug sensitivity, were addressed as a mean of parasite clearance time (MPCT) and analyzed by means of a Microsoft Excel. Item of parasite clearance time (PCT) was employed for odd case.

*P. falciparum*: Those patients with uncomplicated falciparum malaria were treated with a standard regimen of artesunate–fansidar (ARTECOSPE, Guilin Pharmaceutical Co., Ltd. China) combination including 12mg/kg artesunate over 3 d and 25mg/kg fansidar on day 0. The patients were given clinical examinations on days 0, 1, 2, 3, 4, 7, 14, 21& 28. The parasites were counted in thick blood smear as mentioned in *P. vivax* section for each patient on the same days. Therapeutic response to standard regimen of treatment by day 28 of follow up would be classified as early treatment failure (ETF), late treatment failure (LTF) [including late clinical failure (LCF) and late parasitological failure (LPF)] and adequate clinical and parasitological response (ACPR) (12). The results were statistically analyzed with SPSS (ver. 17, Chicago, IL, USA) software based on Independent and Anova tests.

## Results

***P. vivax:*** The patients with *vivax* malaria responded to the regimen of chloroquine in 37.4(±15.9), 40 (±13.8) and 42(±17.7) h for Pakistani, Iranian and Afghani nationalities respectively based on MPCT evaluation. *P. vivax* parasite was found in none of the patients, blood smears after day 3 at follow-up until day 28. MPCT in the blood of females is different from males, but not significant (*P*=0.659, df= 135 and *t*=−0.442) (Table 1). The results showed some differences between Pakistani and Afghani subjects in MPCT, but not between them and Iranian subjects with 37.2 h (1.5d), 42 h (1.7 d) and 40.2 h (1.7d) respectively.

**Table 1:** Minimum and maximum parasitemia of *P. vivax* per microliter of blood according to treatment days, gender and nationalities

***PCT***		***D0***		***D1***		***D2***		***ETF***	***LTF***	***ACPR***	***MPCT h.(day)***
***Nationality Gender***		**Min**	**Max**	**Min**	**Max**	**Min**	**Max**				
**Afghani**	Female	2440	2440	520	520	-	-	-	-	+	*48(2)
	NPCC**		-		1		-				
	Male	1920	4019	47	-	-	-	-	-	+	36(1.5)
	NPCC		1		1		-				
**Pakistani**	Female	79	11200	47	4400	32	358	-	-	+	40(1.6)
	NPCC		-		4		2				
	Male	85	11789	30	8748	80	509	-	-	+	34.3(1.4)
	NPCC		23		12		2				
**Iranian**	Female	57	32636	75	5230	32	827	-	-	+	37.2(1.5)
	NPCC		13		3		4				
	Male	43	46273	16	7962	16	870	-	-	+	43.2(1.8)
	NPCC		26		33		12				

* PCT instead of MPCT due to one case

** Number of parasite cleared cases

***P. falciparum:*** All of the 38-falciparum malaria-infected patients received standard artesunate-fansidar combination treatment after diagnosing the infection as mentioned in materials and methods section. MPCT for the patients with falciparum malaria was calculated as 28(±18.05), 26(±12.03) and 36(±16.9) h for Iranian, Pakistani and Afghani nationalities respectively. There were some hours differences between Afghani and other nationalities (*P*=0.753, df=2) and also between males and females (*P*=0.374, df=36 and *t*=0.901) with 30.7 h (1.3 d) and 24 h (1 day) respectively in the MPCT, but not significant (Tables 2, 3, 4).

**Table 2:** Minimum and maximum parasitemia of *P. vivax* per microliter of blood according to age

***PCT***		***D0***		***D1***		***D2***		***ETF***	***LTF***	***ACPR***	***MPCT h.(day)***
***Age (yr)***		Min	Max	Min	Max	Min	Max				
**2-4**	Parasite	57	32636	39	2936	277	827	-	-	+	39(1.6)
	NPCC*	2		4		2					
**5-9**	Parasite	130	33000	17	2514	16	774	-	-	+	38.4(1.6)
	NPCC	6		2		2					
**10-15**	Parasite	63	17901	120	3792	-	-	-	-	+	33(1.3)
	NPCC	5		3							
**<15**	Parasite	43	11789	16	8748	32	870	-	-	+	40.6(1.7)
	NPCC	50		45		16					

* Number of parasites cleared cases

**Table 3:** Minimum and maximum parasitemia of *P. falciparum* per microliter of blood according to treatment days, gender and nationalities

***PCT***		***D0***		***D1***		***D2***		***D3***		***ETF***	***LTF***	***ACPR***	***MPCT h.(day)***
***Nationality Gender***		Min	Max	Min	Max	Min	Max	Min	Max				
**Afghani**	Female	-	-	-	-	-	-	-	-	-	-	+	-
	NPCC*		-		-		-		-				
	Male	300	7200	624	624	-	-	-	-	-	-	+	36(1.5)
	NPCC		1		1		-		-				
**Pakistani**	Female	2520	2520										**24 (1)
	NPCC		1										
	Male	235	36324	383	383	111	111	-	-	-	-	+	28 (1.2)
	NPCC		16		1		1		-				
**Iranian**	Female	1490	26368	-	-	-	-	-		-	-	+	24(1)
	NPCC		5		-		-		-				
	Male	252	130095	48	48	*	*	600	600	-	-	+	32 (1.3)
	NPCC		10		1		-		1				

* Number of parasites cleared cases

** PCT instead of MPCT due to one case

**Table 4:** Minimum and maximum parasitemia of *P. falciparum* per microliter of blood according to age

***PCT***		***D0***		***D1***		***D2***		***D3***		***ETF***	***LTF***	***ACPR***	***MPCT h.(day)***
***Age (yr)***		Min	Max	Min	Max	Min	Max	Min	Max				
**2-4**	Parasite	26368	26368	-	-	-	-	-	-	-	-	+	*24(1)
	NPCC**	1		-		-		-					
**5-9**	Parasite	3803	25739	383	383	-	-	-	-	-	-	+	32(3.1)
	NPCC	2		1		-		-					
**10-15**	Parasite	3400	3400	-	-	-	-	-	-	-	-	+	*24(1)
	NPCC	1		-		-		-					
**>15**	Parasite	235	130095	48	3626	111	111	600	600	-	-	+	29(2.1)
	NPCC	29		2		1		1					

* PCT instead of MPCT due to one case

** Number of parasite cleared cases

## Discussion

Importance of drug resistance in Plasmodia species particularly in *P. falciparum* stimulates most of the malaria investigators to monitor the efficacy of currently used antimalarial drugs in the malarious areas. Forecasting the time of emerging drug resistance in Plasmodia species prevent most of the hygienic, social and economic damages. This study was proposed to monitor the response of *P. vivax* to chloroquine and *P. falciparum* to artesunate-fansidar combination. Although chloroquine had been used as the first line for treatment of falciparum malaria in Iran up to 2007, due to spreading CQ-resistance in *P. falciparum* strains in Iran from that time the regimen of treatment replaced with currently used artesunate-fansidar combination (13). The first report of drug resistance in *P. falciparum* in Iran was released in 1985 (4). Following the mentioned report several attempts were carried out to evaluate the efficacy of chloroquine and some other antimalarial drugs against *P. falciparum* and *P. vivax* employing either in vivo or *in vitro* tests (11, 14–17) and efficacy of chloroquine against *P. vivax* using in vivo test (6,8,10,11).

Comparing MPCT criterion of *P. vivax* among Iranian, Pakistani and Afghani nationalities showed that there was not any significant difference in clearance time of parasite between patients of the nationalities (Tables 1 and 2). Average MPCT of *P. vivax* in the studied areas was recorded as 40.5 h (1.7d). In a previous study conducted in Bandar-Abbas district of Hormozgan Province in southeastern Iran MPCT of *P. vivax* was calculated as 61.7 h (2.6 d) (11). Moreover, in another study performed in Sistan and Baluchistan Province, near to Pakistan borderline, MPCT of *P. vivax* was recorded as 61.6 h (2.6d) (10). Comparing between the above two results and the results obtained from this study shows some decrease in MPCT of *P. vivax* in the studied areas. Such outcome is supposed to be because of increasing sensitivity of the parasite to chloroquine. Considerable deduction of vivax malaria cases in the areas leads to reduction of chloroquine pressure on the parasites. The process makes the parasites more sensitive to the antimalarial drug. In a cross-sectional study using nested PCR technique, *P. vivax* was still sensitive to chloroquine in southeastern Iran (18).

Among the studied districts, MPCT of *P. vivax* in Mehrestan showed the maximum time with 49.7 h (±2.1 d) and the minimum time in Sarbaz and Saravan with 36 h (1.5d). Interestingly MPCT of the parasite in Chabahar district, a malarious area, decreased from 58 in previous study (10) to 34.7 h in this study. Some reports came out from a number of malarious areas such as Papua New Guinea, Brazil, India, and Colombia about emerging chloroquine-resistant strains of *P. vivax* in the areas (19–23).

## Conclusion

MPCT of *P. vivax* in this study shows that the parasite has become more sensitive to chloroquine than previous years in studied areas, indicating that decrease of *vivax* malaria infection resulted in utilizing less antimalarial drugs against *P. vivax* strains and consequently less pressure of the drugs on the parasites. In this situation, the parasites become more sensitive to the previously used antimalarials due to being omitted many of the resistant mutants.

## References

[1] Ministry of Health and Medical Education Annual report of malaria control department. Tehran, Iran: Centre for Communicable Disease Management; 2015.

[2] Waheed AA Ghanchi NK Rehman KA *Vivax* malaria and chloroquine resistance: a neglected disease as an emerging threat. Malar J. 2015;14:146. 25889875 10.1186/s12936-015-0660-0 PMC4392755

[3] Mebrahtu E Antimalarial drug resistance: In the past, current status and future perspectives. Br J Pharmacol Toxicol. 2015; 6(1):1–15.

[4] Marques MM Costa MR Santana Filho FS *Plasmodium vivax* chloroquine resistance and anemia in the western Brazilian Amazon. Antimicrob Agents Chemother. 2014;58(1):342–7. 24165179 10.1128/AAC.02279-12 PMC3910749

[5] Edrissian GH Shahabi S Preliminary study of the response of *Plasmodium falciparum* to chloroquine in Sistan and Baluchestan province of Iran. Trans R Soc Trop Med Hyg. 1985;79(4):563–4. 3909559 10.1016/0035-9203(85)90101-4

[6] Edrissian GhH Nateghpour M Afshar A Sayedzadeh A Mohsseni Gh Satvat MT Emadi AM Monitoring the Response of *Plasmodium falciparum* and *P. vivax* to Antimalarial Drugs in the Malarious Areas in South-East Iran. Arch Iran Med. 1999; 2(2):61–6.

[7] Edrissian GhH Nateghpour M Afshar A Mohseni GH In vivo monitoring of the response of *falciparum* and *vivax* plasmodia to chloroquine in Bandar Abbas and Kahnoudj, South-East Iran, 1997–1999. Med J Iran Hosp. 2001; 3:30–3.

[8] Hamedi Y Nateghpour M Tan-ariya P Tiensuwan M Silachamroon U Looareesuwan S *Plasmodium vivax* malaria in Southeast Iran in 1999–2001: establishing the response to chloroquine in vitro and in vivo. Southeast Asian J Trop Med Public Health. 2002; 33(3):512–8. 12693585

[9] Raeisi A Ringwald P Safa O Shahbazi A Ranjbar M Keshavarz H Nateghpour M Faraji L Monitoring of the therapeutic efficacy of chloroquine for the treatment of uncomplicated, *Plasmodium falciparum* malaria in Iran. Ann Trop Med Parasitol. 2006;100(1):11–6. 16417708 10.1179/136485906X86220

[10] Nateghpour M Sayedzadeh SA Edrissian GhH Raeisi A Jahantigh A Motevalli Haghi A Mohseni Gh Rahimi A Evaluation of sensitivity of *Plasmodium vivax* to chloroquine. Iran J Publ Health. 2007; 36(3):60–63.

[11] Nateghpour M Edrissian GhH Torabi E Raeisi A Motevalli Haghi A Abed Khojasteh H Gobakhloo N Monitoring the sensitivity of *Plasmodium vivax* and *falciparum* to chloroquine in Bandar-Abbas, Hormozgan province. Tehran Univ Med J. 2009; 67(3):178–183.

[12] World Malaria Report, 2014 Geneva, World Health Organization, 2014. (http://malaria.who.int/campaigns/malaria-day/2014/en/24aApril 2014).

[13] Saebi E Ranjbar M Nabavi M Malaria Treatment Guideline in I.R.Iran. Ministry of Medical Education; 2007.

[14] Edrissian GH Status of the response of *Plasmodium falciparum* to chloroquine and mefloquine in Iran. Trop Geogr Med. 1989;41(4):297–303. 2699684

[15] Edrissian GH Afshar A Sayedzadeh A Mohsseni G Satvat MT Assessment of the response in vivo and in vitro of *Plasmodium falciparum* to sulphadoxine-pyrimethamine in the malarious areas of Iran. J Trop Med Hyg. 1993;96(4):237–40. 8345544

[16] Eskandarian AA Keshavarz H Basco LK Mahboudi F Do mutations in *Plasmodium falciparum* dihydropteroate synthase and dihydrofolate reductase confer resistance to sulfadoxine-pyrimethamine in Iran? Trans R Soc Trop Med Hyg. 2002;96(1):96–8. 11926005 10.1016/s0035-9203(02)90254-3

[17] Sharifi-Sarasiabi K Haghighi A Kazemi B Taghipour N Mojarad EN Gachkar L Molecular surveillance of *Plasmodium vivax* and *Plasmodium falciparum* DHFR mutations in isolates from southern Iran. Rev Inst Med Trop Sao Paulo. 2016; 58:16. 27007559 10.1590/S1678-9946201658016 PMC4804553

[18] Heidari A Keshavarz H Shojaee S Raeisi A Dittrich S In vivo susceptibility of *Plasmodium vivax* to chloroquine in southeastern Iran. Iran J Parasitol. 2012;7(2):8–14. PMC3469182 23109940

[19] Collingon P Chloroquine Resistance in *Plasmodium vivax*. J Infect Dis. 1991; 164(1):222–223. 2056216 10.1093/infdis/164.1.222

[20] Garavelli PL Corti E Chloroquine resistance in *Plasmodium vivax*: the first case in Brazil. Trans R Soc Trop Med Hyg. 1992;86(2):128. 1440766 10.1016/0035-9203(92)90535-k

[21] Schuurkamp GJ Spicer PE Kereu RK Bulungol PK Rieckmann KH Chloroquine-resistant *Plasmodium vivax* in Papua New Guinea. Trans R Soc Trop Med Hyg. 1992; 86(2):121–2. 1440763 10.1016/0035-9203(92)90531-g

[22] Singh RK Emergence of Chloroquine-resistant *vivax* malaria in south Bihar (India). Trans R Soc Trop Med Hyg. 2000; 94(3):327. 10975013

[23] Soto J Toledo J Gutierrez P Luzz M Llinas N Cedeño N Dunne M Berman J *Plasmodium vivax* clinically resistant to chloroquine in Colombia. Am J Trop Med Hyg. 2001;65(2):90–3. 11508397 10.4269/ajtmh.2001.65.90

